# *Arabidopsis U2AF65* Regulates Flowering Time and the Growth of Pollen Tubes

**DOI:** 10.3389/fpls.2019.00569

**Published:** 2019-05-03

**Authors:** Hyo-Young Park, Hee Tae Lee, Jeong Hwan Lee, Jeong-Kook Kim

**Affiliations:** ^1^Division of Life Sciences, Korea University, Seoul, South Korea; ^2^Division of Life Science, Chonbuk National University, Jeonju, South Korea

**Keywords:** *AtU2AF65a*, *AtU2AF65b*, flowering time, male gametophyte development, pollen tube growth, premRNA splicing

## Abstract

During pre-mRNA splicing, U2 small nuclear ribonucleoprotein auxiliary factor 65 (U2AF65) interacts with U2AF35 and splicing factor 1 (SF1), allowing for the recognition of the 3′-splice site by the ternary complex. The functional characterization of *U2AF65* homologs has not been performed in *Arabidopsis thaliana* yet. Here, we show that normal plant development, including floral transition, and male gametophyte development, requires two *Arabidopsis U2AF65* isoforms (*AtU2AF65a* and *AtU2AF65b*). Loss-of-function mutants of these two isoforms displayed opposite flowering phenotypes: *atu2af65a* mutants showed late flowering, whereas *atu2af65b* mutants were characterized by slightly early flowering, as compared to that in the wild-type (Col-0) plants. These abnormal flowering phenotypes were well-correlated with the expression patterns of the flowering time genes such as *FLOWERING LOCUS C* (*FLC*) and *FLOWERING LOCUS T* (*FT*). However, the two *atu2af65* mutants did not display any morphological abnormalities or alterations in abiotic stress tests. Double mutation of the *AtU2AF65a* and *AtU2AF65b* genes resulted in non-viable seeds due to defective male gametophyte. *In vitro* pollen germination test revealed that mutations in both *AtU2AF65a* and *AtU2AF65b* genes significantly impaired pollen tube growth. Collectively, our findings suggest that two protein isoforms of AtU2AF65 are differentially involved in regulating flowering time and display a redundant role in pollen tube growth.

## Introduction

In eukaryotic cells, pre-messenger RNA (pre-mRNA) splicing is important for processes such as expression of intron-containing genes, remodeling of protein-protein interaction networks, and regulation of transcript abundance (Nilsen and Graveley, 2010). Spliceosomes, consisting of five small nuclear ribonucleoproteins (snRNPs) and numerous non-snRNPs, mediate these key cellular functions (Wahl et al., 2009). The U2 snRNP auxiliary factor (U2AF) is one such non-snRNP component. In mammalian cells, U2AF is composed of two subunits, U2AF65 and U2AF35, representing the large and the small subunits, respectively. During assembly of the spliceosome, U2AF65 directly binds to the polypyrimidine (Py) tracts upon interaction with SF1 through its third RNA recognition motif (RRM) (Rain et al., 1998; Thickman et al., 2006). Meanwhile, U2AF35 binds to the 3′-splice sites and may also promote the binding of U2AF65 to the Py tract through its interaction with the U2AF65 and serine-arginine (SR)-rich proteins (Zuo and Maniatis, 1996). These molecular interactions are essential for the initial recognition of the intronic 5′- and 3′-splice sites by the spliceosome. Although plant introns contain neither conserved branch-point sequences nor Py tracts (Luehrsen et al., 1994; Simpson and Filipowicz, 1996), similar interactions found in mammals may also be present in plants. Two U2AF65 homologs purified from wild tobacco (*Nicotiana plumbaginifolia*) can rescue the *in vitro* splicing of adenovirus pre-mRNA in the extracts of U2AF factor-deficient HeLa cells (Domon et al., 1998). In addition, the *Arabidopsis* genome has *U2AF35*, *U2AF65*, and *SF1* homologs (Wang and Brendel, 2006; Jang et al., 2014), and *Arabidopsis* U2AF65 (AtU2AF65) and SF1 (AtSF1) proteins interact with their rice homologs (Jang et al., 2014; Park et al., 2017). These results suggest that the interactions among splicing factors during intron 3′-splice site recognition is conserved in plants; however, plant introns do not contain conserved regions such as the branch point sequences and Py tracts at the 3′-splice proximal sites found in mammalians.

Although the detailed splicing mechanism is poorly understood in plants, some components of the plant spliceosome play essential roles in plant growth and development, such as in the flowering time. Mutations in *AtU2AF35a* or *AtU2AF35b* result in pleiotropic phenotypes including altered flowering time and abnormal morphological defects in leaves, flowers, and siliques (Wang and Brendel, 2006). A loss of *AtSF1* activity also causes pleiotropic developmental defects such as early flowering and hypersensitivity to abscisic acid (ABA) (Jang et al., 2014). Furthermore, these mutations in spliceosomal components result in altered splicing patterns of specific genes in plants. For instance, altered *AtU2AF35* expression generates novel splicing isoforms of the *FCA* pre-mRNA due to the splicing of non-canonical introns (Wang and Brendel, 2006). The mutation of *AtSF1* affects the alternative splicing pattern of the heat shock transcription factor *HsfA2* (Jang et al., 2014; Lee et al., 2017). These results suggest that lesions in spliceosomal components affect alternative splicing of a specific set of genes. Recent reports have also shown that ambient temperature shifts lead to the alternative splicing of splicing-related genes (Verhage et al., 2017) and the cyclin-dependent kinase G1 (CDKG1) affects the alternative splicing of *AtU2AF65a* in response to the changes in ambient temperature (Cavallari et al., 2018). These results suggest that the components of the spliceosome may be the target of ambient temperature-induced alternative splicing, thereby coordinating plant growth and development under continuously fluctuating temperature conditions.

The mutations in spliceosomal components also result in defects in female- or male-gametophytes. Loss-of-function mutations in genes encoding components of snRNPs such as the U2 snRNP-specific proteins ATROPOS (ATO)/Prp9 (Moll et al., 2008) and SF3b130 (Aki et al., 2011), the U5 snRNP-specific protein GAMETOPHYTIC FACTOR 1 (GFA1)/CLOTHO (CLO)/VAJRA (VAJ)/Snu114 (Moll et al., 2008; Yagi et al., 2009), and the U4/U6 snRNP-specific protein LACHESIS (LIS)/Prp4 (Gross-Hardt et al., 2007; Volz et al., 2012) lead to abolished or impaired genetic transmission through the female gametophyte. Mutations in the genes for the DEAH-box RNA-dependent ATPases *Prp22*/*ROOT INITIATION DEFECTIVE1* (*RID1*) and *Prp16*/*CLUMSY VEIN* (*CUV*) cause defects in the female and male gametophyte, respectively (Ohtani et al., 2013; Tsugeki et al., 2015). Notably, mutants of *ATO*, *SF3b130*, *GFA1*/*CLO*/*VAJ*, and *RID1* show less severe defects in male gametophyte development. However, a lesion in the *SHOOT REDIFFERENTIATION DEFECTIVE 2* (*SRD2*) gene, causing defective expression of all the snRNAs, does not result in abnormal transmission through the male gametophyte (Onouchi et al., 2000; Ohtani and Sugiyama, 2005; Onodera et al., 2008). These results suggest that loss-of-activity mutations of spliceosomal components differentially affect male and female gametophyte development.

Although *U2AF65* genes have been identified in plants such as *Arabidopsis*, rice, and tobacco (Gniadkowski et al., 1996; Domon et al., 1998), the function of *U2AF65* in plants has not yet been reported. Four *U2AF65* homologs are predicted in the *Arabidopsis* genome and only two of these (hereafter defined as *AtU2AF65a* and *AtU2AF65b*) have a U2AF homology motif (UHM), i.e., the third RNA recognition motif (RRM3) (Wang and Brendel, 2006; Jang et al., 2014). Here we show that defects in *AtU2AF65a* and *AtU2AF65b* may cause abnormal flowering time and pollen tube growth during the male gametophyte development.

## Materials and Methods

### Plant Materials and Growth Conditions

All mutants used in this study were *Arabidopsis* plants in the Columbia (Col-0) background, except for *fy-1* (L*er*) (Simpson et al., 2003; Henderson et al., 2005). Various T-DNA alleles of *AtU2AF65a* (SALK_075828, SALK_128420C, SALK_142561, and SALK_144790) and *AtU2AF65b* (SALK_055049C and SALK_104128) were obtained from the *Arabidopsis* Biological Resource Center (ABRC) (Alonso et al., 2003). The T-DNA homozygous lines and the double mutants used in this study were verified by DNA-based PCR genotyping using gene-specific and T-DNA specific primers. The oligonucleotide sequences used for genotyping are provided in Supplementary Table S1. The plants were grown in Sunshine Mix 5 (Sun Gro Horticulture, United States) or Murashige and Skoog (MS) medium at 23 or 16°C under long-day (LD) [16/8 h (light/dark)] or short-day (SD) [8/16 h (light/dark)] conditions with light supplied at an intensity of 120 μmol m^-2^ s^-1^.

### Plasmid Construction

To check the β-glucuronidase (GUS) expression patterns driven under *AtU2AF35* and *AtU2AF65* promoter, we generated the *pAtU2AF35a*_0.9_ *_kb_::GUS*, *pAtU2AF35b*_0.7_ *_kb_::GUS*, *pAtU2AF65a*_1.3_ *_kb_::GUS*, and *pAtU2AF65b*_1.5_ *_kb_::GUS* constructs. For *AtU2AF35a*, the genomic region from 876 nucleotides (nt) before the ATG start codon was cloned, as previously described (Wang and Brendel, 2006). For *AtU2AF35b*, the genomic region from 700 nt before the start codon was newly cloned, because the previous report has shown that the promoter region of different lengths (555 and 982 nt) have similar GUS staining patterns (Wang and Brendel, 2006). For *AtU2AF65a*, the cloned region is from 1,300 nt before the start codon and includes the 5′-untranslated region (UTR) region of *AtU2AF65a*, and the 3′-UTR region and part of the exon of *At4G36700*. For *AtU2AF65b*, we had tried to clone constructs with the promoter region of different lengths, which begin 1,500 nt before the start codon, because this gene has an intergenic region (approximately 6,800 nt). However, we were not able to detect GUS staining-positive clones from any of the promoter-GUS constructs. We are going to characterize this unusual trait of the *AtU2AF65b* promoter in the near future. We amplified these promoter regions of each gene using specific primers and cloned them into the pGWB433 vector (gift from Tsuyoshi Nakagawa at the Research Institute of Molecular Genetics, Shimane University, Japan). To make the *pAtU2AF65a_890__bp_::AtU2AF65a* construct, the full-length coding sequence (CDS) of *AtU2AF65a* and the genomic region from 890 nt before the start codon including the 5′-UTR region of *AtU2AF65a* and the 3′-UTR region of *At4G36700* were amplified and cloned into a pCHF3 vector. The reason we used the shorter *AtU2AF65a* promoter for the complementation test is that we wanted to find out the shorter promoter required for *AtU2AF65a*’s function. The construct with 890 nt promoter construct was our first and single one in order to check its usefulness for the complementation test. To produce the *p35S::AtU2AF65b* construct, the full-length CDS of *AtU2AF65b* was amplified and cloned into a pCHF3 vector. The resulting recombinant plasmid was sequenced to verify the absence of PCR errors during amplification. The oligonucleotide sequences used in this study are listed in Supplementary Table S1.

### Generation of Transgenic Plants and Measurement of Flowering Time

Transgenic plants were generated using the floral dip method with minor modifications (Weigel and Glazebrook, 2002). The *Agrobacterium tumefaciens* strain GV3101, harboring the gene constructs, was infiltrated into the *AtU2AF65* mutant backgrounds. Transgenic seedlings were first selected for pCHF3 vector content using kanamycin and then verified by PCR-based genotyping. A number of T_1_ seedlings between 20 and 30 were analyzed for each construct.

To assess the flowering time, the total numbers of rosette and cauline leaves of at least five or six independent transgenic lines (at least 16 individual plants per transgenic line) were counted in the T_2_ or T_3_ generation (when we checked flowering time in T_3_ generation, all experiments were repeated three times). To determine whether the flowering time of the transgenic plants significantly differed from that of the wild-type (Col-0) plants, the data were analyzed using the SPSS software version 12.0 (Michaels, 2009). The error bars indicate standard error of the mean (SEM) of three biological replicates consisting of independently harvested samples. The leaf number ratio (16°C/23°C, LNR) under LD conditions was used as an indicator of temperature-responsive flowering (Lee et al., 2007). A hypothetical temperature-sensitive plant produces a different total number of leaves at 23 and 16°C; thus, its LNR is near 2.0.

### Germination Assays Under Hormone Treatment or Abiotic Stress Conditions

The sensitivity of the plants to hormones and abiotic treatments was determined as previously described (Hugouvieux et al., 2001). Seeds were sown on half-strength MS medium containing 1% sucrose, and the MS medium was maintained at 4°C for 2 days in the dark, after which it was transferred to a normal growth chamber kept at 23°C under LD conditions at a light intensity of 120 μmol m**^-^**^2^ s**^-^**^1^. Seed germination was analyzed in MS medium supplemented with various concentrations of ABA, NaCl, and mannitol. The germination rate of the seeds was measured at the indicated days. All experiments were repeated three times.

### RNA Expression Analyses

Thirty micrograms of total RNA was used for northern blot analysis as described previously (Jang et al., 2014), and ^32^P-labeled *AtU2AF65a* and *AtU2AF65b* cDNA, which are approximately 500 nt fragment of CDSs starting from the ATG start codon in both *AtU2AF65a* and *AtU2AF65b*, were used as probes due to the unique nucleotide sequences for each gene. For reverse transcription-polymerase chain reaction (RT-PCR) and real-time quantitative PCR (RT-qPCR) analyses, total RNA was extracted from a variety of tissues or from 8-day-old whole seedlings of *Arabidopsis* plants using QIAzol Lysis reagent (Qiagen, Germany). The RNA quality was determined using a Nanodrop ND-2000 spectrophotometer (Nanodrop Technologies, Waltham, MA, United States), and only the high-quality RNA samples (A260/A230 > 2.0 and A260/A280 > 1.8) were used for subsequent experiments. The first-strand complementary DNA (cDNA) was synthesized from 1 μg of total RNA in accordance with the manufacturer’s instructions (Invitrogen, United States). The RT-qPCR analysis was carried out in 384-well plates with a LightCycler 480 (Roche Applied Science, United States) using SYBR green. RT-qPCR experiments were carried out using Roche SYBR Green Master mixture (Roche Applied Science, United States). In accordance with the best established RT-qPCR practices (Hong et al., 2010), *PP2AA3* was included as a reference gene due to its stable expression in *Arabidopsis*. All RT-PCR and RT-qPCR experiments were performed in three biological replicates consisting of independently harvested samples with three technical replicates each. The error bars indicate the SEM of three biological replicates consisting of independently harvested samples. The oligonucleotide sequences used for expression analysis are provided in Supplementary Table S1. Changes in gene expression were calculated via the ΔΔ_CT_ method (Ramakers et al., 2003). For GUS expression analysis, GUS staining was carried out as described by Chen et al. (1998).

### Characterization of Pollen Grains and Pollen Tubes

Pollen viability was assayed as described by Alexander (1969). For *in vitro* pollen tube growth assay, the pollen grains from mutant and wild-type flowers were plated onto the surface of agar plates and cultured at 23°C with a relative humidity of 100% for 3 h and then examined using a light microscope (OLYMPUS model BX-51). The detailed procedure has been previously reported (Jiang et al., 2005). All experiments were repeated three times. To determine whether the proportion data such as pollen morphology and pollen tube length significantly differ between mutants and wild-type (Col-0) plants, the data were analyzed by χ^2^ test.

## Results

### Isolation and Characterization of the Single Mutants of *AtU2AF65a* and *AtU2AF65b*

**Figure 1:**
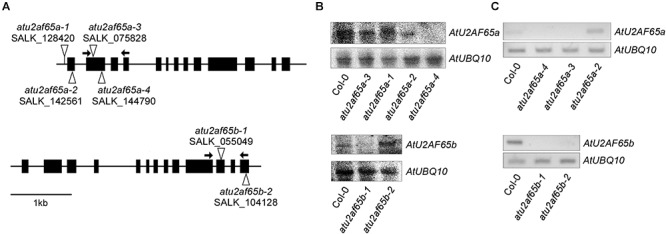
Characterization of T-DNA insertion alleles of *AtU2AF65a* and *AtU2AF65b*. **(A)** Schematic diagrams of T-DNA insertions in *atu2af65a* and *atu2af65b* mutants. The triangles and the black boxes indicate the T-DNA insertions and the exons of *AtU2AF65a* and *AtU2AF65b* genes, respectively. The arrows indicate the primers used for the RT-PCR analysis to detect the transcripts from each T-DNA mutant allele. **(B)** Northern blot analysis of several T-DNA alleles in *atu2af65a* and *atu2af65b* mutants. The expression levels of the *AtU2AF65a* and *AtU2AF65b* genes were checked in 10-day-old SALK T-DNA allele lines and wild-type (Col-0) plants grown at 23°C under LD conditions. The location of probes used in northern blot analysis was as follows: approximately 500 nucleotides from the start codon of the coding sequence (CDS) of both *AtU2AF65a* and *AtU2AF65b*. *AtUBQ10* expression served as a loading control. **(C)** RT-PCR analysis of several T-DNA alleles in *atu2af65a* and *atu2af65b* mutants.

**Figure 2:**
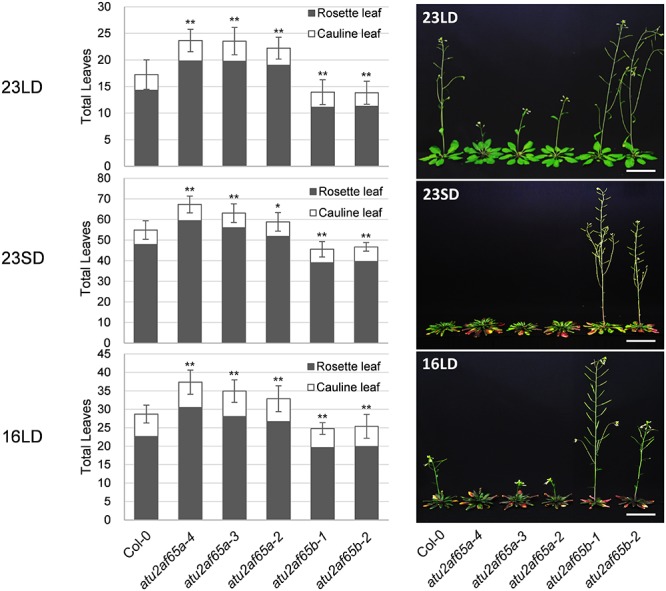
Flowering phenotypes of *atu2af65a* and *atu2af65b* mutants under different growth conditions. Plants were grown at 23°C under long-day (LD) condition (23LD), at 23°C under short-day (SD) condition (23SD) or at 16°C under long-day (LD) condition (16LD). The number of total leaves (rosette plus cauline leaves) shown in the bar graphs represents the flowering phenotype of each plant. As a control, the wild-type (Col-0) plants were used. The photos were taken upon *atu2af65b* mutant flowering. The error bars indicate the standard error of the mean (SEM) of three biological replicates consisting of independent samples. The asterisks denote a significant difference in flowering time between transgenic and wild-type (Col-0) plants (Student’s *t*-test, ^∗^*P* < 0.05, ^∗∗^*P* < 0.01). Scale bars 3 cm.

We have recently revealed that AtSF1 and AtU2AF65 interact with each other and that the alteration of *AtSF1* activity affects the flowering time (Jang et al., 2014; Lee et al., 2017). Therefore, we analyzed the phenotypes of the T-DNA alleles of *AtU2AF65a* and *AtU2AF65b* to study their biological function. We obtained four T-DNA alleles (SALK_128420C, SALK_142561, SALK_075828, and SALK_144790) for *AtU2AF65a* and two T-DNA alleles (SALK_055049C and SALK_104128) for *AtU2AF65b* to isolate homozygous T-DNA insertional mutants for each gene. PCR genotyping and sequencing analyses confirmed the T-DNA insertion sites of each T-DNA allele (Figure 1A,B). They were renamed *atu2af65a-1* (SALK_128420C), *atu2af65a-2* (SALK_142561), *atu2af65a-3* (SALK_075828), *atu2af65a-4* (SALK_144790), *atu2af65b-1* (SALK_055049C), and *atu2af65b-2* (SALK_104128), respectively. Northern blot analysis revealed that *AtU2AF65a* and *AtU2AF65b* expression did not occur in *atu2af65a-4* and *atu2af65b-1* mutants, respectively (Figure 1A,B), whereas the other *AtU2AF65a* and *AtU2AF65b* T-DNA alleles displayed significantly lower or similar expression than the wild-type (Col-0) plants, suggesting that they were knock-down mutants. We also performed RT-PCR analyses using primer sets that spanned the T-DNA insertion sites in three *atu2af65a* and two *atu2af65b* mutant alleles. PCR fragments of the expected size were detected in wild-type (Col-0) plants, but no amplification was observed for the regions spanning the T-DNA insertion sites in these mutant alleles (Figure 1C). Based on these observations, we used the *atu2af65a-4* and *atu2af65b-1* knock-out mutants for further studies. As shown in Figure 2, the single *atu2af65a* and *atu2af65b* mutants did not show major defects in growth and development, except for flowering. The flowering time of *atu2af65a-4* mutants was delayed at 23°C under LD conditions as compared to that of wild-type (Col-0) plants, whereas that of *atu2af65b-1* mutants was slightly accelerated [*atu2af65a-4*, *atu2af65b-1*, and wild-type (Col-0) plants = 23.7, 14.0, and 17.2 leaves, respectively)]. These flowering phenotypes of *atu2af65a* and *atu2af65b* mutants were consistent with the findings (Verhage et al., 2017; Xiong et al., 2019). Notably, the expression of the *AtU2AF65a* gene (*pAtU2AF65a_890__bp_::AtU2AF65a*) in the *atu2af65a-4* mutant background rescued the mutant phenotype, whereas *AtU2AF65b* overexpression (*p35S::AtU2AF65b*) in the *atu2af65b-1* mutant background was not able to restore the wild-type (Col-0) phenotype (Supplementary Figures S1, S2). In order to establish whether different light or temperature conditions affect the flowering time, we further examined the impact on this parameter of plant incubation either at 23°C under SD or at 16°C under LD conditions. The flowering time phenotypes of these mutants were not significantly affected by the change in light/temperature conditions and the relative ratios remained substantially unchanged. For instance, *atu2af65a-4* plants produced more leaves at 16°C (37.9 leaves) than at 23°C (23.2 leaves) (LNR = 1.6). Under same conditions, wild-type (Col-0) plants exhibited a delayed flowering time [16°C (28.7 leaves) vs. 23°C (16.1 leaves); LNR = 1.8]. This indicated that the flowering of *atu2af65a-4* plants was sensitive to differences in temperature. More specifically, at 23°C under SD conditions, the leaf counts were 67.3, 45.6, and 54.9 for *atu2af65a-4*, *atu2af65b-1*, and wild-type (Col-0) plants, respectively. Furthermore, vernalization treatment showed that both the *atu2af65* mutants and wild-type (Col-0) plants responded similarly to this condition (Supplementary Figure S3). However, the *atu2af65a-4* and *atu2af65b-1* single mutants did not respond to several abiotic stresses including exposure to various concentrations of ABA, NaCl, and mannitol (Supplementary Figure S4). These flowering time data indicated that *AtU2AF65a* and *AtU2AF65b* differentially affected the flowering time control in *Arabidopsis*.

### Expression Patterns of *AtU2AF65a* and *AtU2AF65b*

**Figure 3:**
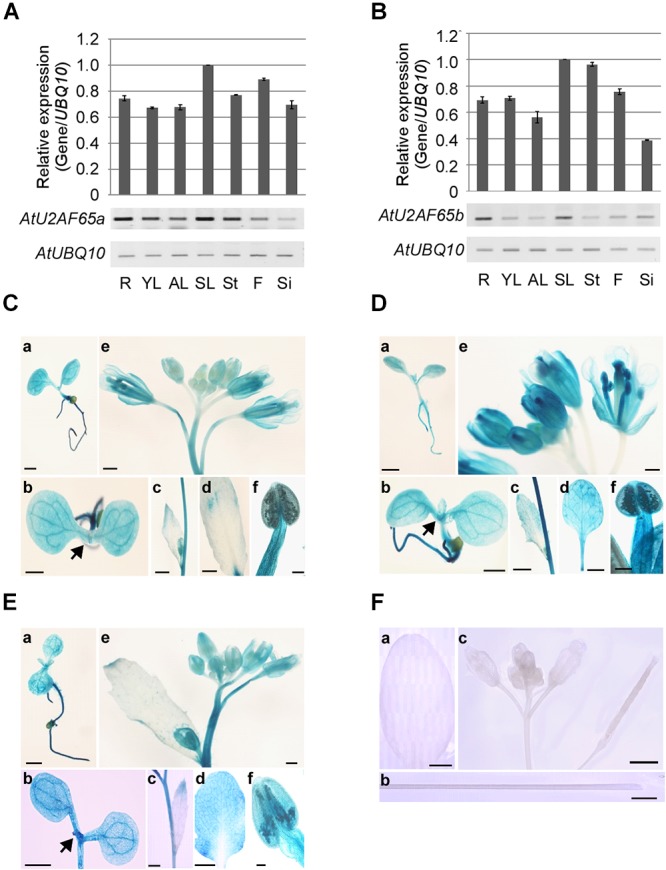
Expression patterns of *AtU2AF65a* and *AtU2AF65b* genes. **(A,B)** RT-PCR analysis of *AtU2AF65a* and *AtU2AF65b* expression in a variety of tissues [roots (R), young leaves (YL), adult leaves (AL), senescent leaves (SL), stems (St), flowers (F), and siliques (Si)] of wild-type (Col-0) plants grown at 23°C under LD conditions. The expression level of each *AtU2AF65* gene in SL was set to 1. *AtUBQ10* expression served as a loading control. The error bars indicate the SEM of three biological replicates consisting of independently harvested samples, with technical replicates for each sample. **(C–F)** Histochemical GUS staining in *pAtU2AF35a*_0.9_ *_kb_::GUS* **(C)**, *pAtU2AF35b*_0.7_ *_kb_::GUS* **(D)**, *pAtU2AF65a*_1.3_ *_kb_::GUS* **(E)**, and *GUS* **(F)** plants under LD conditions. GUS staining was performed in a variety of tissues [seedlings **(a,b)**, cauline leaves **(c)**, adult leaves **(d)**, inflorescences **(e)**, and stigmas **(f)**]. Arrows indicate shoot apex regions. GUS expression was not detected in transgenic plants harboring *GUS* constructs without a promoter region [**F**, adult leaves **(a)**, stems **(b)**, and inflorescences **(c)**]. Scale bars 100 μm. Note that the GUS expression patterns of *pAtU2AF35b*_0.7_ *_kb_::GUS* were similar to those of *pAtU2AF35b*_550_ *_bp_::GUS* or *pAtU2AF35b*_982_ *_bp_::GUS*, as previously described (Wang and Brendel, 2006).

We analyzed the expression patterns of endogenous *AtU2AF65a* and *AtU2AF65b* by RT-PCR analysis. The *AtU2AF65a* and *AtU2AF65b* transcripts were abundantly expressed in a variety of tissues (Figure 3A,B) and their expression levels were very similar in most tissues, such as vegetative tissues and flowers. However, the expression level of *AtU2AF65a* was approximately twofold higher than that of *AtU2AF65b* in siliques. These ubiquitous expression patterns were similar to those of other splicing factor genes such as *AtSF1* and *AtU2AF35* in *Arabidopsis* (Wang and Brendel, 2006; Jang et al., 2014).

In order to investigate the spatial expression patterns of *AtU2AF65a* and *AtU2AF65b* in more detail, we analyzed β-glucuronidase (GUS) activity in transgenic *pAtU2AF65a_1.3__kb_::GUS* and *pAtU2AF65b_1.5__kb_::GUS* plants, harboring regions of about 1.3 and 1.5 kb upstream of the ATG of *AtU2AF65*, respectively. We also made *pAtU2AF35a_0.9__kb_::GUS* and *pAtU2AF35b_0.7__kb_::GUS* plants harboring about 0.9 or 0.7 kb regions upstream of the *AtU2AF35* ATG, to compare the GUS staining patterns between *AtU2AF65* and *AtU2AF35*. We were able to detect GUS activities in all the transgenic plants except for the *pAtU2AF65b::GUS* plants, because our attempt to clone the promoter of the *AtU2AF65b* gene was not successful. The spatial expression patterns of the GUS activities were very similar in most tissues. Strong GUS activities were detected in all parts of the young seedlings including shoot apex regions (Figure 3Ca,Cb,Da,Db,Ea,Eb). Older leaves also showed intense GUS staining in the *pAtU2AF65a*_1.3_ *_kb_::GUS* and *pAtU2AF35b*_0.7_ *_kb_::GUS* plants (Figure 3Dc,Dd,Ec,Ed). Particularly high GUS expression was observed in some parts of the flowers such as sepals, stamens, anthers, pollen, and stigma (Figure 3Ce,Cf,De,Df,Ee,Ef). Petals showed weak GUS expression. However, GUS activities were not observed in the tissues of the transgenic plants expressing only GUS constructs (Figure 3F). In general, the GUS staining patterns were consistent with the results of RT-PCR analysis (Figure 3A,B) and with the findings in *pAtSF1::GUS* and *pAtU2AF35::GUS* plants (Wang and Brendel, 2006; Jang et al., 2014).

### Expression of Flowering Time Genes in *atu2af65* Mutants

**Figure 4:**
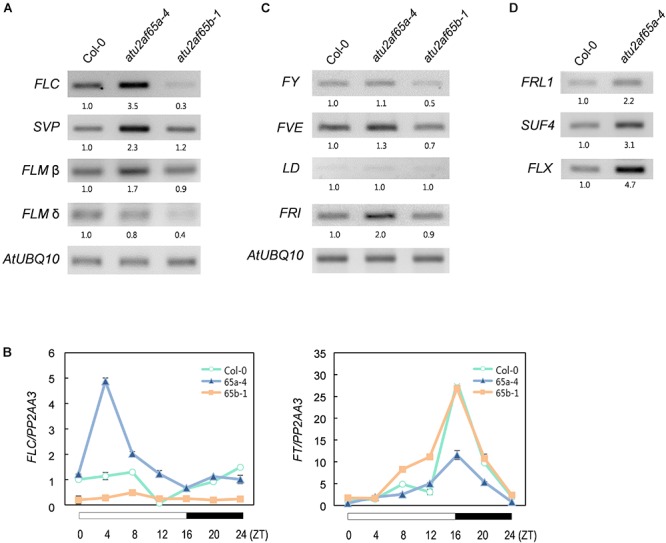
Expression of flowering time genes in the *atu2af65a* and *atu2af65b* mutants. **(A)** RT-PCR analysis of *FLC*, *FLM*, and *SVP* expression in the *atu2af65a-4* and *atu2af65b-1* mutants grown at 23°C under LD conditions. *AtUBQ10* expression served as a loading control. The numbers below the gels denote the fold change relative to wild-type (Col-0) plants. **(B)** RT-qPCR analysis of *FLC* and *FT* expression in the *atu2af65a-4* and *atu2af65b-1* mutants grown at 23°C under LD conditions. *PP2AA3* expression served as a loading control. The error bars indicate the SEM of three biological replicates consisting of independently harvested samples, with technical replicates for each sample. **(C)** RT-PCR analysis of *FY*, *FVE*, *LD*, and *FRI* expression in the *atu2af65a-4* and *atu2af65b-1* mutants grown at 23°C under LD conditions. **(D)** RT-PCR analysis of *FRL1*, *SUF4*, and *FLX* expression in the *atu2af65a-4* and *atu2af65b-1* mutants grown at 23°C under LD conditions. *AtUBQ10* expression was shown in **(C)**.

To examine whether the observed changes in the flowering phenotype of the *atu2af65a-4* and *atu2af65b-1* mutants were correlated with altered expression of flowering time genes, we performed RT-PCR and RT-qPCR analyses of the 8-day-old whole seedlings grown at 23°C under LD conditions. The expression of *FLOWERING LOCUS C* (*FLC*), *SHORT VEGETATIVE PHASE* (*SVP*), and *FLOWERING LOCUS M*-β (*FLM*-β) significantly increased in the *atu2af65a-4* mutants, whereas the expression of only *FLC* was selectively decreased in the *atu2af65b-1* mutants (Figure 4A). However, the expression of *FLM-δ* as another *FLM* spliced form was not altered in the *atu2af65a-4* mutants. Moreover, the expression levels of *FLOWERING LOCUS T* (*FT*), which is a major downstream target of *FLC*, *SVP*, and *FLM* (Lee et al., 2013; Pose et al., 2013), were significantly altered (Figure 4B). We also observed changes in the spliced or unspliced isoforms of the *FLC* transcripts in the *atu2af65a-4* and *atu2af65b-1* mutants (Supplementary Figure S5), indicating that the altered alternative splicing of these *FLC* transcripts affected the changes of major *FLC* transcripts in these mutants. The reduced expression levels and altered splicing patterns of *FLC* observed in *atu2af65b-1* mutants were consistent with the recent report (Xiong et al., 2019). However, we did not find significant changes in the alternative splicing of *FCA* or *SVP* transcripts (Wang and Brendel, 2006).

We next investigated the expression of negative and positive regulators of *FLC* in *atu2af65* mutants. To this end, the transcriptional profiles of *FLOWERING CONTROL LOCUS A* (*FCA*), *FY*, *FVE*, *LUMINIDEPENDENS* (*LD*) (negative *FLC* regulators) as well as *FRIGIDA* (*FRI*) (a positive *FLC* regulator) were addressed (Ratcliffe et al., 2001). Although minor alterations in *FY* and *FVE* expression were observed in the *atu2af65a-4* or *atu2af65b-1* mutants (Figure 4C), these changes were too modest to explain the altered flowering phenotype shown by the *atu2af65* mutants (Figure 2). Interestingly, *FRI* expression was significantly increased only in the *atu2af65a-4* mutants. Because the genetic background of *atu2af65a-4* mutants is Col-0, which includes a weak *FRI* allele (Gazzani et al., 2003), we further checked the expression levels of three other *FLC*-specific regulators (Choi et al., 2011), i.e., *FRIGIDA LIKE1* (*FRL1*), *SUPPRESSOR OF FRIGIDA4* (*SUF4*), and *FLC EXPRESSOR* (*FLX*). Their strong expression also observed in the *atu2af65a-4* mutants (Figure 4D). Collectively, these results suggest that the late flowering phenotype of *atu2af65a-4* mutants is likely due to increased *FLC* activity in an *FRI* complex-dependent manner, whereas the early flowering phenotype of *atu2af65b-1* mutants is likely caused by decreased *FLC* activity in an *FRI* complex-independent manner.

### The *atu2af65* Mutations Are Defective in Male Gametophyte Function

**Figure 5:**
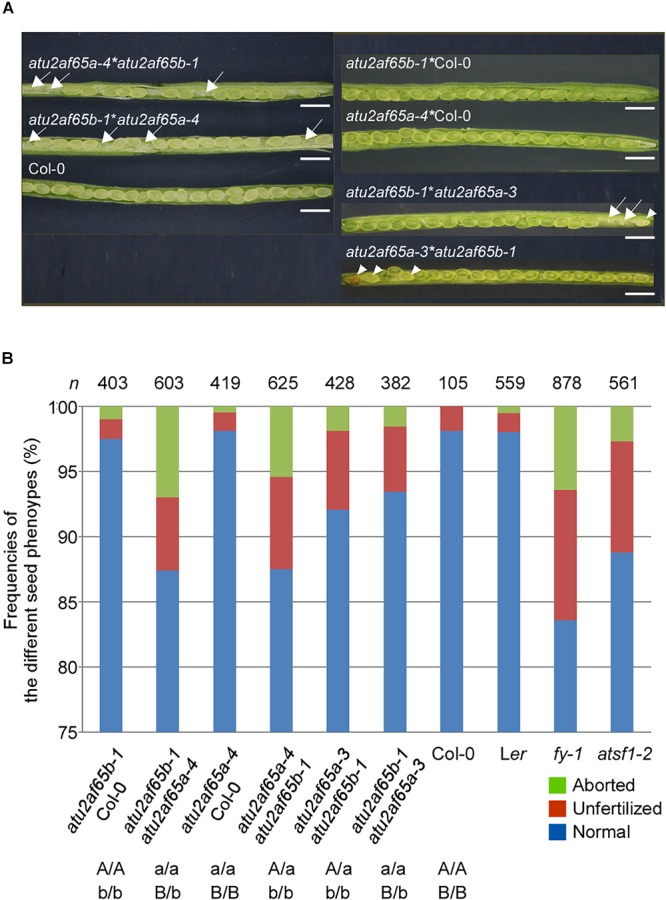
Seed developmental defects in siliques of various combinations of *atu2af65a* and *atu2af65b* mutants. **(A)** Seed phenotype of dissected siliques from various *atu2af65a atu2af65b* heterozygous double mutants in the F_2_ progeny. The plants obtained from the crosses between *atu2af65a-4* or *atu2af65a-3*, and *atu2af65b-1* single mutants as male parents and wild-type plants as female parents were used as controls. The arrows and arrow heads indicate the unfertilized ovules and aborted seeds, respectively. The classification of seed phenotypes in siliques has been described previously (Zhou et al., 2013). Scale bars 1.5 mm. **(B)** A histogram showing the fractions of normal and aborted seeds, and unfertilized ovules from various *atu2af65a atu2af65b* heterozygous double mutants in the F_3_ progeny. The genotypes of F_3_ progenies are indicated below the histogram. Other splicing factor mutants such as *fy-1* and *atsf1-2* were used to compare the seed developmental defects of *atu2af65a atu2af65b* heterozygous double mutants. L*er* and Col-0 plants were used as controls for *fy-1* mutants and other plants. *n* denotes the numbers of siliques used for the analysis of seed phenotype.

Because the *atu2af65a* and *atu2af65b* single mutants did not display noticeable defective phenotypes, except for flowering time (Figure 2), we decided to investigate the phenotype of *atu2af65a atu2af65b* double mutants. To generate these double mutants, the *atu2af65a-4* homozygous mutants, either as male or female parents, were crossed with the *atu2af65b-1* homozygous mutants. Genetic crossing was also performed using the *atu2af65a-3* homozygous mutants, as a source of knock-down allele. The resulting F_1_ progeny [+/*atu2af65a-4*;+/*atu2af65b-1* (*65A*/*65a*;*65B*/*65b*) or +/*atu2af65a-3*;+/*atu2af65b-1*] was subsequently allowed to self-pollinate and the genotypes of the F_2_ progeny were determined by PCR-based genotyping. No progeny of homozygous double mutants [*atu2af65a-4*/*atu2af65a-4*;*atu2af65b-1*/*atu2af65b-1* (*65a*/*65a*;*65b*/*65b*) or *atu2af65a-3*/*atu2af65a-3*;*atu2af65b-1*/*atu2af65b-1*] was identified, suggesting lethality of the homozygous double mutation as well as redundancy of *AtU2AF65a* and *AtU2AF65b* for plant growth and development. Only the heterozygous plants for one mutant allele and homozygous for the other [+/*atu2af65a-4*;*atu2af65b-1*/*atu2af65b-1* (*65A*/*65a*;*65b*/*65b*) and *atu2af65a-4*/*atu2af65a-4*;+/*atu2af65b-1* (*65a*/*65a*;*65B*/*65b*), or +/*atu2af65a-3*;*atu2af65b-1*/*atu2af65b-1* and *atu2af65a-3*/*atu2af65a-3*;+/*atu2af65b-1*] were found in the F_2_ progeny. When these heterozygous double mutants were self-fertilized, no plants with the homozygous double mutants were identified in the progeny. Figure 5A shows the seed phenotype observed in the siliques of various combinations of mutants in the F_3_ progeny. Dissection of siliques from heterozygous double mutants [+/*atu2af65a-4*;*atu2af65b-1*/*atu2af65b-1* (*65A*/*65a*;*65b*/*65b*) and *atu2af65a-4*/*atu2af65a-4*;+/*atu2af65b-1* (*65a*/*65a*;*65B*/*65b*)] revealed a seed abortion rate (the frequencies of aborted seeds and unfertilized ovules in the analyzed seeds) of about 12.5%, whereas control plants [F_3_ progenies produced by crossing *atu2af65a-4* or *atu2af65b-1* homozygous mutants with wild-type (Col-0) plants] showed an abortion rate of about 2.2%, similar to that of wild-type (Col-0) plants (about 1.9%) (Figure 5B). However, the heterozygous double mutants [+/*atu2af65a-3*;*atu2af65b-1*/*atu2af65b-1* (*65A*/*65a*;*65b*/*65b*) and *atu2af65a-3*/*atu2af65a-3*;+/*atu2af65b-1* (*65a*/*65a*;*65B*/*65b*)] using the *atu2af65a-3* homozygous mutants as a knock-down allele showed a seed abortion rate of about 7.3%, suggesting that the residual activity of *AtU2AF65a* in the *atu2af65a-3* mutants could affect seed development. Interestingly, *atsf1-2* mutants showed a seed abortion rate of about 11.3%, which was similar with that observed in heterozygous double mutants [+/*atu2af65a-4*;*atu2af65b-1*/*atu2af65b-1* (*65A*/*65a*;*65b*/*65b*) and *atu2af65a-4*/*atu2af65a-4*;+/*atu2af65b-1* (*65a*/*65a*;*65B*/*65b*)] (Figure 5B). Previously characterized *fy-1* mutants with defects in seed development (Henderson et al., 2005) also showed seed abortion rates of about 16.4%. However, silique morphology of heterozygous double mutants was not significantly affected and no embryo or seedling lethals were observed. These data indicated that the combinations of *atu2af65a* and *atu2af65b* null alleles lead to defects in genetic transmission through the male or female gametes.

To investigate the impact of *atu2af65a* and *atu2af65b* mutations on male and female gametophytic functions, we performed reciprocal crosses between *atu2af65a-4*/*atu2af65a-4*;+/*atu2af65b-1* (*65a*/*65a*;*65B*/*65b*) and *atu2af65a-4*/*atu2af65a-4*;+/+ (*65a*/*65a*;*65B*/*65B*), and tested transmission of the *atu2af65b-1* allele in an *atu2af65a-4* background, through both the male and the female haploid stage. Each genotype of progeny was confirmed by DNA-based PCR genotyping. *atu2af65b-1* allele transmission through the male gametophyte was completely blocked [ratio of *atu2af65a-4*/*atu2af65a-4*;+/+ (*65a*/*65a*;*65B*/*65B*): *atu2af65a-4*/*atu2af65a-4*;+/*atu2af65b-1* (*65a*/*65a*;*65B*/*65b*) = 0], whereas *atu2af65b-1* allele transmission through the female gametophyte was normal [ratio of *atu2af65a-4*/*atu2af65a-4*;+/+ (*65a*/*65a*;*65B*/*65B*): *atu2af65a-4*/*atu2af65a-4*;+/*atu2af65b-1* (*65a*/*65a*;*65B*/*65b*) = 1:1.2] (Table 1). Also, transmission of the *atu2af65a-4* allele through the male gametophyte in reciprocal crosses between +/*atu2af65a*;*atu2af65b*/*atu2af65b* (*65A*/*65a*;*65b*/*65b*) was completely interrupted (Supplementary Table S2). This data indicate that mutations in *AtU2AF65a* and *AtU2AF65b* affect the genetic transmission through the male gametes.

**Table 1:** Transmission of the *atu2af65a*;*atu2af65b* genotype through the male and female gametophytes.

Cross			65a/65a;65B/65B	*65a*/*65a*;*65B*/*65b*	Number of progeny genotyped	TE female (%)	TE male (%)
Female	Male					
*65a*/*65a*;*65B*/*65b*	*65a*/*65a*;*65B*/*65B*	22	26	48	118	NA
*65a*/*65a*;*65B*/*65B*	*65a*/*65a*;*65B*/*65b*	48	0	48	NA	0

*NA, not applicable; TE, transmission efficiency calculated according to Howden et al. (1998)*.

### The *atu2af65* Mutations Redundantly Impair Pollen Tube Growth

**Figure 6:**
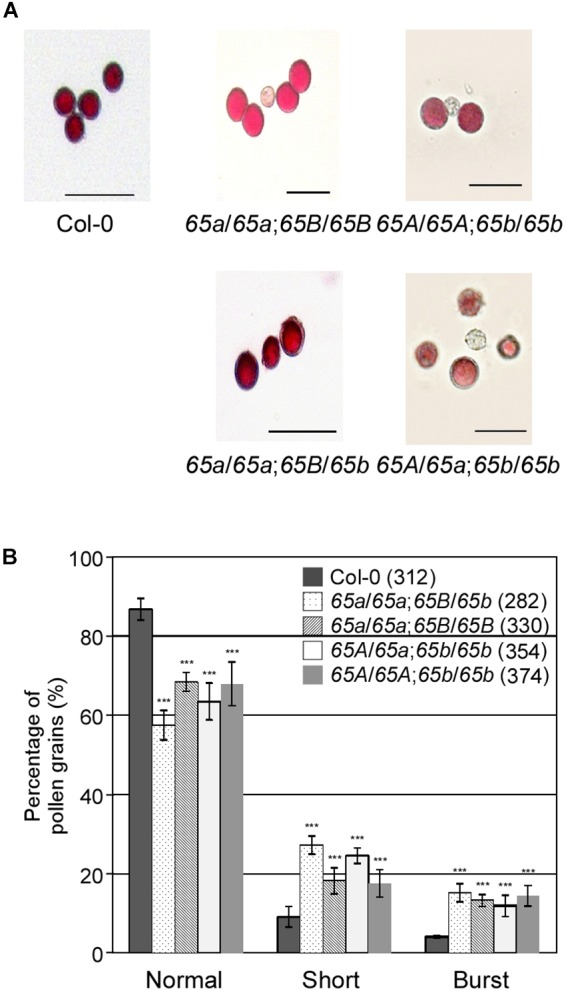
Pollen morphology in the F_2_ progeny containing the *atu2af65a* or *atu2af65b* mutant alleles. **(A)** Pollen morphology from wild-type (Col-0, *65A*/*65A*;*65B*/*65B*), *atu2af65a-4*/*atu2af65a-4*;+/+ (*65a*/*65a*;*65B*/*65B*), +/+; *atu2af65b-1*/*atu2af65b-1* (*65A*/*65A*;*65b*/*65b*), *atu2af65a-4*/*atu2af65a-4*;+/*atu2af65b-1* (*65a*/*65a*;*65B*/*65b*), +/*atu2af65a-4*/*atu2af65b-1*/*atu2af65b-1* (*65A*/*65a*;*65b*/*65b*) plants. The *atu2af65a* mutant plants with the (*65a*/*65a*;*65B*/*65b*) genotype or without *atu2af65b* mutant alleles (*65a*/*65a*;*65B*/*65B*) and the *AtU2AF65b* mutant plants with (*65A*/*65a*;*65b*/*65b*) genotype or without *AtU2AF65a* mutant alleles (*65A*/*65A*;*65b*/*65b*) were selected from the F_2_ progeny population. Scale bars 50 μm. **(B)** Shape of pollen grains shown in panel **(A)**. The shape of pollen was categorized into three groups (normal, short, and burst). The average of three biological replicates is shown in the histogram. Numbers in parenthesis denote the total number of pollen grains in three biological replicates of each genotype used for this analysis (approximately 110 pollen grains was used for each biological replicate). The error bars indicate the SEM of three biological replicates consisting of independently harvested samples. The asterisks denote a significant difference in pollen morphology between mutants and wild-type (Col-0) plants (χ^2^ test, ^∗∗∗^*P* < 0.001).

To investigate how the mutations in *AtU2AF65a* and *AtU2AF65b* affect male gametophytic function, we phenotypically analyzed the *AtU2AF65* mutants used as male parts in gametophytic function test. Alexander staining results showed that the pollen grains from *atu2af65a-4*/*atu2af65a-4*;+/+ (*65a*/*65a*;*65B*/*65B*), +/+;*atu2af65b-1*/*atu2af65b-1* (*65A*/*65A*;*65b*/*65b*), *atu2af65a-4*/*atu2af65a-4*;+/*atu2af65b-1* (*65a*/*65a*;*65B*/*65b*), and +/*atu2af65a-4*;*atu2af65b-1*/*atu2af65b-1* (*65A*/*65a*;*65b*/*65b*) plants were morphologically indistinguishable from wild-type (Col-0) pollen grains (Figure 6A), indicating that the *atu2af65* mutations did not affect pollen grain formation. However, the fractions of approximately 31.6 and 32.1% of the pollen grains were short or burst in the single mutants [*atu2af65a-4*/*atu2af65a-4*;+/+ (*65a*/*65a*;*65B*/*65B*) and +/+; *atu2af65b-1*/*atu2af65b-1* (*65A*/*65A*;*65b*/*65b*)], respectively, whereas in the wild-type (Col-0) plants, this fraction was approximately 13.3% (Figure 6B). Furthermore, approximately 42.5% of the pollen grains was short or burst in *atu2af65a-4*/*atu2af65a-4*;+/*atu2af65b-1* (*65a*/*65a*;*65B*/*65b*) plants. Notably, the *atu2af65a-4*/*atu2af65a-4*;+/*atu2af65b-1* (*65a*/*65a*;*65B*/*65b*) pollen grains displayed a more profound impairment than the *atu2af65a-4*/*atu2af65a-4*;+/+ (*65a*/*65a*;*65B*/*65B*) pollen grains. This data indicated that the mutant pollens are highly unstable under the employed *in vitro* conditions.

**Figure 7:**
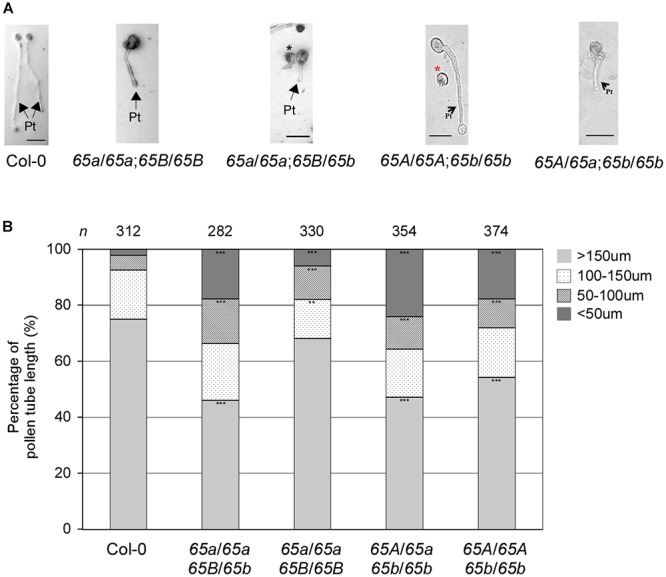
*In vitro* pollen tube growth defects in the F_2_ progeny containing the *AtU2AF65a* or *AtU2AF65b* mutant alleles. **(A)** Morphology of pollen tube growth from wild-type (Col-0, *65A*/*65A*;*65B*/*65B*), *atu2af65a-4*/*atu2af65a-4*;+/+ (*65a*/*65a*;*65B*/*65B*), +/+; *atu2af65b-1*/*atu2af65b-1* (*65A*/*65A*;*65b*/*65b*), *atu2af65a-4*/*atu2af65a-4*;+/*atu2af65b-1* (*65a*/*65a*;*65B*/*65b*), +/*atu2af65a-4*;*atu2af65b-1*/*atu2af65b-1* (*65A*/*65a*;*65b*/*65b*) plants. Pollen grains were germinated *in vitro* as in Figure 6. Arrows with Pt indicate pollen tubes germinated from pollens and asterisks (black and red) denote abnormal pollen grains. Scale bars 50 μm. **(B)** The length of pollen tube. Pollen tube length was measured after a 24-h incubation. The pollen tube length was categorized into four groups (shorter than 50 μm: <50 μm, between 50 and 100 μm: 50–100 μm, between 100 and 150 μm: 100–150 μm, longer than 150 μm: >150 μm). The average of three biological replicates is shown in the histogram. *n* indicates the total number of pollen grains in three biological replicates of each genotype used for this analysis (approximately 110 pollen grains was used for each biological replicate). The asterisks denote a significant difference in pollen morphology between mutants and wild-type (Col-0) plants (χ^2^ test, ^∗∗^*P* < 0.01, ^∗∗∗^*P* < 0.001).

Next, we investigated the *in vitro* germination of pollen grains from the F_2_ progeny of various combinations of the *atu2af65a* and *atu2af65b* mutants. The pollen grains from both mutants germinated efficiently under the described *in vitro* conditions (data not shown), but the resulting pollen tubes were severely abnormal (Figure 7A). We also examined the length of pollen tubes after germination for 24 h. The mean length of the mutant pollen tubes was significantly reduced in comparison with that of the wild-type (Col-0) pollen tubes (Figure 7B): only 68.2 and 54.3% of the *atu2af65a-4*/*atu2af65a-4*;+/+ (*65a*/*65a*;*65B*/*65B*) and +/+; *atu2af65b-1*/*atu2af65b-1* (*65A*/*65A*;*65b*/*65b*) pollen tubes, respectively, were > 150 μm in length, whereas 75.0% of the wild-type (Col-0) pollen tubes were above 150 μm in length. Furthermore, the fraction of pollen tubes above 150 μm in length was smaller in the heterozygous double mutants [46.1 and 47.2% for the *atu2af65a-4*/*atu2af65a-4*;+/*atu2af65b-1* (*65a*/*65a*;*65B*/*65b*) and +/*atu2af65a-4*;*atu2af65b-1*/*atu2af65b-1* (*65A*/*65a*;*65b*/*65b*) mutants, respectively] than in the single mutants. Collectively, our results suggested that the mutations in *AtU2AF65a* and *AtU2AF65b* significantly impair the growth of pollen tubes, which may affect pollen tube growth during fertilization.

## Discussion

Pre-mRNA splicing plays an essential role in various growth and developmental processes in plants, but as yet little is known about the function of many of the spliceosomal components. Here, we provided evidence that two isoforms of *AtU2AF65* are important for flowering and for the growth of pollen tubes during male gametophyte development.

Since plant introns do not contain the conserved branch point sequences and Py tracts at the 3′-splice proximal sites found in mammalians (Wahl et al., 2009), the mechanism of 3′-splice site recognition in plants may differ. Our previous findings, however, showed that AtU2AF65 proteins physically interact with AtU2AF35 and AtSF1 proteins *in vitro* and *in planta* (Jang et al., 2014; Park et al., 2017), as already known for yeasts and metazoans. Therefore, the initial step consisting in 3′-splice site recognition may be functionally similar in mammalians and plants, despite the above mentioned structural differences in plant introns. This notion is supported by the finding that two tobacco U2AF65 homologs (NpU2AF65a and NpU2AF65b) can splice adenoviral pre-mRNA in HeLa cell extracts depleted of endogenous U2AF factors (Domon et al., 1998). However, some differences in the mechanism of intron recognition may occur in plants. Firstly, several copies of *U2AF* genes such as *U2AF65* and *U2AF35* exist in nearly all higher plant genomes (Wang and Brendel, 2006; Jang et al., 2014). Secondly, plant SF1 homologs have an RRM domain, which is not found in their fungal and metazoan counterparts (Worden et al., 2009). Thirdly, neither the branch point nor the 3′-splice site regions significantly contribute to the interaction between RNA and U2AF65 homologs, as is the case with tobacco (Domon et al., 1998). Finally, we observed differential binding affinities of AtU2AF65a and AtU2AF65b for AtU2AF35 proteins (Park et al., 2017). These differences can account for the diversity and flexibility of molecular interactions between various components of the spliceosome in the initial recognition of the 3′-splice site in plants.

Several lines of evidence suggest that mutations in both *AtU2AF65a* and *AtU2AF65b* may result in the changes in flowering time. Our and other groups have previously reported that the AtU2AF65 and AtU2AF35 proteins interact (Park et al., 2017) and both T-DNA mutation in *AtU2AF35a* as well as RNA interference against *AtU2AF35b* cause late flowering phenotypes (Wang and Brendel, 2006; Jang et al., 2014). Moreover, we found that the expression patterns of *AtU2AF35* and *AtU2AF65* were similar (Figure 3; Wang and Brendel, 2006). For instance, RT-PCR analyses showed that *AtU2AF35* and *AtU2AF65* expression was observed in all vegetative and reproductive tissues; however, the abundance of their transcripts was different in the tissues tested. The GUS assays also revealed that the staining patterns of *AtU2AF65a* promoter activity were similar to those of *AtU2AF35a* and *AtU2AF35b* in all the tissues analyzed; however, their GUS staining intensities were slightly different. However, T-DNA alleles of *AtU2AF65a* were associated with a late flowering phenotype, whereas T-DNA alleles of *AtU2AF65b* resulted in early flowering (Figure 2). Due to this partial inconsistency, the phenotype of *atu2af65b* mutants should be interpreted with caution, especially since the overexpression of *AtU2AF65b* in the *atu2af65b* mutant background was not able to rescue the mutant phenotype (Supplementary Figure S2). It is therefore difficult to infer the *AtU2AF65b* function from the results obtained with the T-DNA mutant alleles. One possible explanation is that AtU2AF65b does not affect the flowering time via a dominant negative effect when it is ectopically overexpressed, because overexpressed AtU2AF65b could interrupt the formation of functional complexes, which does not objectively reflect the flowering processes in wild-type plants. However, we cannot exclude the possibility that *AtU2AF65b* regulates flowering time under specific conditions such as abiotic or biotic stresses. A recent report showed that the expression of *AtU2AF65b* is increased by ABA and *AtU2AF65b* modulated ABA-mediated flowering (Xiong et al., 2019). Furthermore, another T-allele (WiscDsLox321F12) of *AtU2AF65b* has an early flowering phenotype under LD and SD conditions, which was confirmed our observations (Figure 2).

*AtU2AF65a* and *AtU2AF65b* appear to differentially control *FLC* expression, and this might result in the opposite flowering phenotypes observed. However, considering the expression patterns of flowering time-related genes, including positive and negative regulators of *FLC*, in the *atu2af65a* and *atu2af65b* mutants (Figure 4), and the effect of vernalization on the flowering time in these mutants (Supplementary Figure S3), it is likely that *AtU2AF65a* and *AtU2AF65b* control flowering time by modulating *FLC* expression in *FRI* complex–dependent and –independent manners, respectively. Similarly, the mutations in *CAP-BINDING PROTEIN 80* (*CBP80*)/*ABA HYPERSENSITIVE 1* (*ABH1*) affect flowering time via *FRI*-mediated *FLC* regulation (Bezerra et al., 2004). However, because the genetic background (Col-0) of the *atu2af65a* mutants includes a weak *FRI* allele (Gazzani et al., 2003), further investigations are needed to ascertain whether an altered *AtU2AF65* function may have an impact on *FLC* expression in the *FRI FLC* background. Furthermore, genome-wide RNA-seq analysis in the *fri FLC* and *FRI FLC* backgrounds is required to identify the general or specific effect of *atu2af65* mutation on splicing regulation in flowering. These information would result in a better understanding of the control exerted by *AtU2AF65* on flowering time.

Our study strongly suggests redundant regulation of pollen tube growth by different *AtU2AF65* isoforms during male gametophyte development. Firstly, GUS expression patterns of *AtU2AF65* were strongly observed in pollen (Figure 3). Secondly, *atu2af65a atu2af65b* heterozygous double mutants (*65A*/*65a*;*65b*/*65b* and *65a*/*65a*;*65B*/*65b*) showed higher seed abortion rates (about 12.5%) than single *atu2af65* mutants (about 2.2%) (Figure 5B). Thirdly, reciprocal cross test revealed that *atu2af65b-1* allele transmission through the male gametophyte was completely interrupted (Table 1 and Supplementary Table S2). Finally, the instability of pollen shape and impaired pollen tube growth observed in *atu2af65a atu2af65b* heterozygous double mutants (*65A*/*65a*;*65b*/*65b* and *65a*/*65a*;*65B*/*65b*) were more pronounced than those induced by the single *atu2af65* mutants (Figure 6, 7).

The importance of splicing factors during male gametophyte development has been reported by several research groups (Tsukaya et al., 2013). For instance, the mutations in *ATO*, *SF3b130*, *GFA1*/*CLO*/*VAJ*, and *RID1* cause defects in male gametophyte development (Moll et al., 2008; Yagi et al., 2009; Ohtani et al., 2013). Since these genes are involved in cell wall synthesis, cytoskeletal functions, metabolism, cellular transport, signaling, and secretion, all important elements for pollen tube growth, one possible scenario is that *AtU2AF65* is involved in the pre-mRNA splicing of a specific set of genes for pollen tube development. This notion is supported by previous evidence showing that mutations in genes involved in a number of processes, including potassium transport, membrane trafficking, carbohydrate metabolism, signaling cascades, and cell wall functions, affect pollen germination or pollen tube growth (Mouline et al., 2002; Johnson et al., 2004; Lalanne et al., 2004; Lobstein et al., 2004; Jiang et al., 2005). Notably, as the U2AF65-associated protein (UAP56) plays an important role in the nuclear export of mRNAs in mammals (Fleckner et al., 1997; Gatfield et al., 2001), another possible scenario is that *AtU2AF65* affects the export of nuclear mRNAs for pollen tube development. This hypothesis is supported by the observation that *Arabidopsis* UAP56 interacts with mRNA export factors such as the RNA and export factor-binding proteins (REF)/ALY2 and MODIFIER OF SNC1 (MOS11) (Kammel et al., 2013). Therefore, *AtU2AF65* may affect the splicing or nuclear export of the pre-mRNAs of a specific set of genes, thereby ensuring normal pollen tube stability and growth during male gametophyte development. However, direct evidence in support of this hypothesis is lacking and further work is needed to definitely establish the role of the *AtU2AF65* in male gametophyte development.

## Author Contributions

J-KK conceived and designed the research. H-YP and HL conducted the experiments. JL and J-KK analyzed the data. JL and J-KK wrote the manuscript. All authors read and approved the manuscript.

## Conflict of Interest Statement

The authors declare that the research was conducted in the absence of any commercial or financial relationships that could be construed as a potential conflict of interest.
