# Supplementary material for: Arabidopsis U2AF65 Regulates Flowering Time and the Growth of Pollen Tubes
Source: Front Plant Sci. 2019 May 3;10:569. doi: 10.3389/fpls.2019.00569 (PMC6510283; doi:10.3389/fpls.2019.00569)
Supplement: Supplementary file 1 [file Table_1.DOCX]

Supplementary Material

***Arabidopsis U2AF65* Regulates Flowering Time and the Growth of Pollen Tubes**

**Hyo-Young Park^1^, Hee Tae Lee^1^, Jeong Hwan Lee^2*^, and Jeong-Kook Kim^1*^**

^1^Division of Life Sciences, Korea University, Republic of Korea. ^2^Division of Life Sciences, Chonbuk National University, Republic of Korea

**^*^Correspondence:** Dr. Jeong Hwan Lee ([jhwanlee90@jbnu.ac.kr](mailto:jhwanlee90@jbnu.ac.kr)) & Dr. Jeong-Kook Kim **(**[jkkim@korea.ac.kr](mailto:jkkim@korea.ac.kr))

**Table S1. Oligonucleotides used in this study**

| **Gene** | **Purpose** | **Primers (5' to 3')** | **Direction** |
| --- | --- | --- | --- |
| *FLM-β* | RT-PCR | CATGCTGATGAACTTAGAGCCT | F |
|  |  | CAGCAACGTATTCTTTCCCAT | R |
| *FLM-δ* | RT-PCR | GATAGAAGCGCTGTTCAAGC | F |
|  |  | CAGCAACGTATTCTTTCCCAT | R |
| *FT* | RT-PCR/RT-qPCR | CTTGGCAGGCAAACAGTGTATGCAC | F |
|  |  | GCCACTCTCCCTCTGACAATTGTAGA | R |
| *SOC1* | RT-PCR/RT-qPCR | AAACGAGAAGCTCTCTGAAAAG | F |
|  |  | AAGAACAAGGTAACCCAATGAAC | R |
| *FLC* | RT-PCR/RT-qPCR | GCCAAGAAGACCGAACTCAT | F |
|  |  | TTTGTCCAGCAGGTGACATC | R |
| *SVP* | RT-PCR | CAAGGACTTGACATTGAAGAGCTTCA | F |
|  |  | CTGATCTCACTCATAATCTTGTCAC | R |
| *SVP.1* | RT-PCR | CTGTAGCTCCAGCATGAAGGAA | F |
|  |  | TGAAGTTCGCTGATCTCACTCATA | R |
| *SVP.2* | RT-PCR | GAAAACTGTTCGACATGAAGGAAG | F |
|  |  | TGAAGTTCGCTGATCTCACTCATA | R |
| *FCA* | RT-PCR | CCCTTGCAGAATCCTTATGCTTAT | F |
|  |  | ATCTCCCCAAGAACTCATGTGGA | R |
| *FY* | RT-PCR | ATGCGGCAGTCGTCAGCTT | F |
|  |  | GACGTCTTATCACGGGCTC | R |
| *FVE* | RT-PCR | ACGAAGCAGCAGCAGTGTCT | F |
|  |  | TTGAAACCCAACCAAACCGTC | R |
| *LD* | RT-PCR | TTGACAATCCGAAATTAGGTAACC | F |
|  |  | ACTCAAAAGACAAGAGTGAGGAA | R |
| *FRI* | RT-PCR | CAAGGAGCCAGCGAAGTTTG | F |
|  |  | GTGGAATGCATATTGAAGCTCTT | R |
| *FRL1* | RT-PCR | TTTTAGCAGTCAAATTCATGTA | F |
|  |  | ATCAGAACCTTGAATCAGAGTT | R |
| *SUF4* | RT-PCR | AGGAATTCCACCCCATGTCTTGA | F |
|  |  | GCGATAGACAGACGAATCTCAG | R |
| *FLX* | RT-PCR | GCGTGAGCTCGAACGGTTAC | F |
|  |  | TAGTAACCTTCACCATGAGCGC | R |
| *PP2AA3* | RT-qPCR | GCGGTTGTGGAGAACATGATACG | F |
|  |  | GAACCAAACACAATTCGTTGCTG | R |
| *AtUBQ10* | RT-PCR | CACCATTGACAACGTGAAGG | F |
|  |  | ACGCAGGACCAAGTGAAGAG | R |
| 65a_GUS | GUS | AAGCTTGGAACGGCAACTAC | F |
|  |  | GGATCCATCCCTGCTTCGG | R |
| SALK_075828LP | Genotyping | CACAAAGACCACCAAACAACC | F |
| SALK_075828RP |  | GCGAAAAGATGTCTGAATTCG | R |
| SALK_142561LP | Genotyping | AATGGGAAATGGAAGGTGAAG | F |
| SALK_142561RP |  | GTCTCTCTTTCATGGTCACGC | R |
| SALK_144790LP | Genotyping | CCTAGTTTACCATCCCTGG | F |
| SALK_144790RP |  | GCGAAAAGATGTCTGAATTCG | R |
| SALK_055049cLP | Genotyping | GAATTCGTCTTCGTAGTCGCCTT | F |
| SALK_055049cRP |  | TCAGGCAATGACTCAACAG | R |
| SALK_104128 LP | Genotyping | TTCGAAGGGATATGCATTCTG | F |
| SALK_104128 RP |  | TGAGAGCAATTGCACATGTTC | R |
| *65A NLS2* | Genotyping for 65AOX(or endoA) rescue genotyping | AAAGCAGGCTTCAGTCGTCGATCT | F |
|  |  | AGAAAGCTGGGTCTCTATCATCTCTC | R |
| *65B NLS2* | Genotyping for 65BOX(or endoB) rescue genotyping | AAAGCAGGCTTCACAGGCGACGGT | F |
|  |  | AGAAAGCTGGGTCTCTGTCTCGATC | R |
| *65A full* | RT-PCR | AAAGCAGGCTTCATGTCTGAATTC | F |
|  |  | AGAAAGCTGGGTCGGCTCCATAA | R |
| *65B full* | RT-PCR | AAAGCAGGCTTCATGATGAGTTACG | F |
|  |  | AGAAAGCTGGGTCGTCTTCGTAGT | R |
| *65A E2_E4* | RT-PCR for genotyping | TGAAAGAGAGACATCAAGAAGTAAA | F |
|  |  | CTAACATTGCAGAGGCTGGAG | R |
| *65B E10_E13* | RT-PCR for genotyping | CCGAGCAAGAAGAAGTCTTACT | F |
|  |  | CATACTTGTCTTCGGGGTAATAC | R |
| *FLC In2* | RT-PCR | TTATGCATACCGCAATTTTCATAGC | F |
|  |  | ATTTAAGGTGGCTAATTAAGTAGTG | R |

**Table S2. Transmission of the *atu2af65a*;*atu2af65b* genotype through the male and female gametophytes**

| Cross |  | *65A*/*65A*  *65b*/*65b* | *65A*/*65a*  *65b*/*65b* | Number of  progeny genotyped | TE female (%) | TE male (%) |
| --- | --- | --- | --- | --- | --- | --- |
| Female | Male |  |  |  |  |  |
| *65A*/*65a*  *65b*/*65b* | *65A*/*65A*  *65b*/*65b* | 27 | 21 | 48 | 78 | NA |
| *65A*/*65A*  *65b*/*65b* | *65A*/*65a*  *65b*/*65b* | 48 | 0 | 48 | NA | 0 |

NA, not applicable; TE, transmission efficiency calculated according to Howden *et al.* (1998).


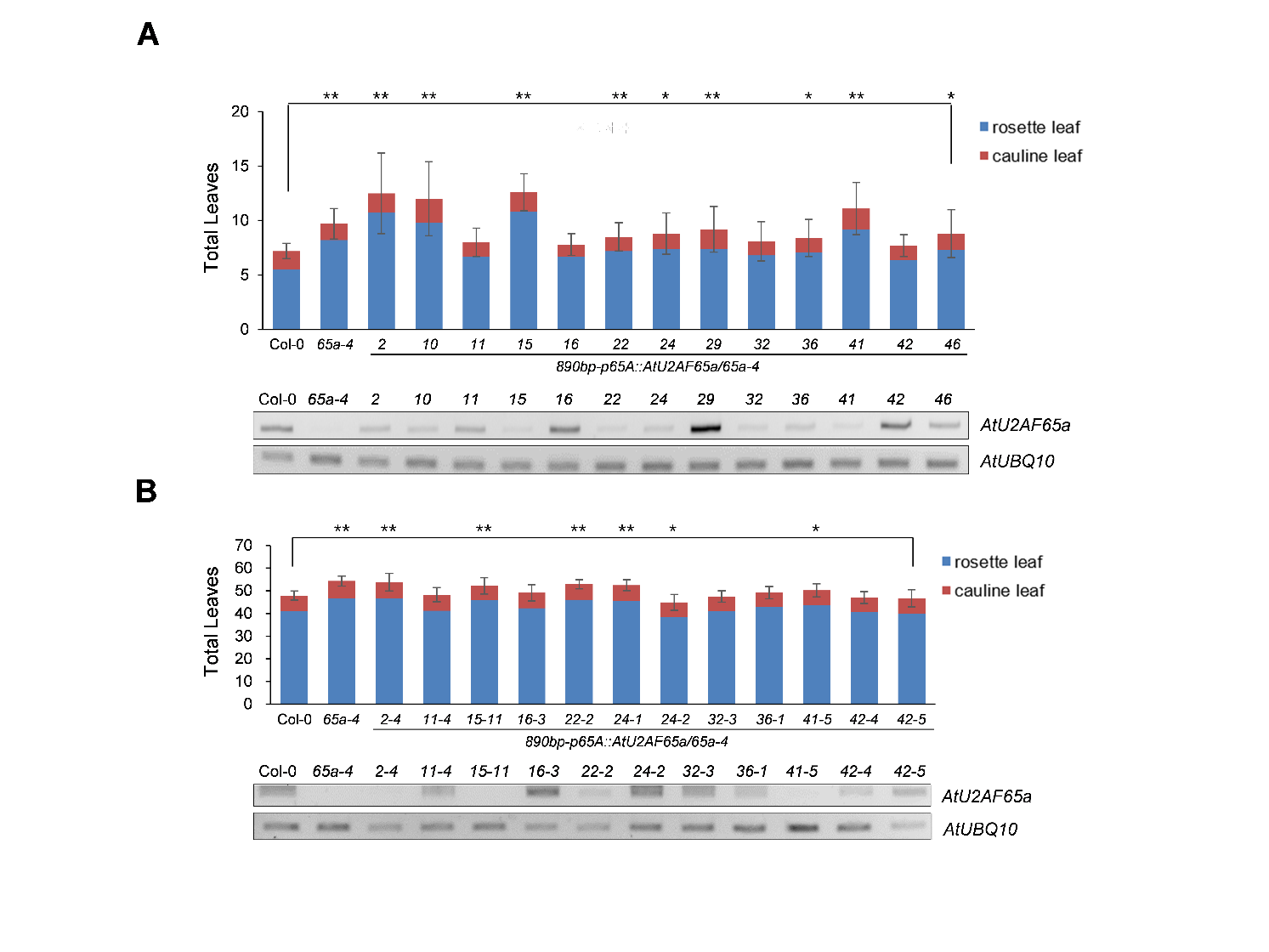


**Figure S1. Flowering phenotype of the rescued transgenic plants of the *atu2af65a* mutants.** The full-length cDNA of *AtU2AF65a* was introduced into the *atu2af65a-4* mutant plants using its own promoter (*pAtU2AF65a_890bp_::AtU2AF65a* in *atu2af65a-4*) to restore the abnormal flowering phenotype of the mutant plants. Plants were grown at 23^o^C under long-day (LD) (**A**) and short-day (SD) (**B**) conditions. Flowering time of the transgenic plants in the T_2_ generation was measured. Total leaves (rosette leaves plus cauline leaves) shown in the bar graphs represented the flowering phenotypes of each plants. As a control, the wild-type (Col-0) plants were used. Error bars indicate the standard deviation. The asterisks denote a significant difference in the flowering time of the transgenic plants compared with that of the wild-type (Col-0) plants (Student’s *t*-test, ^*^*P* < 0.05, ^**^*P* < 0.01). Noted that the transgenic lines with *AtU2AF65a* expression complemented the late flowering phenotype of *atu2af65a-4* mutants. Note that the promoter of *AtU2AF65a* used for complementation test is the genomic region from 890 nt before the ATG start codon including the 5’–untranslated region (UTR) region of *AtU2AF65a* and the 3’–UTR region of *At4G36700*.


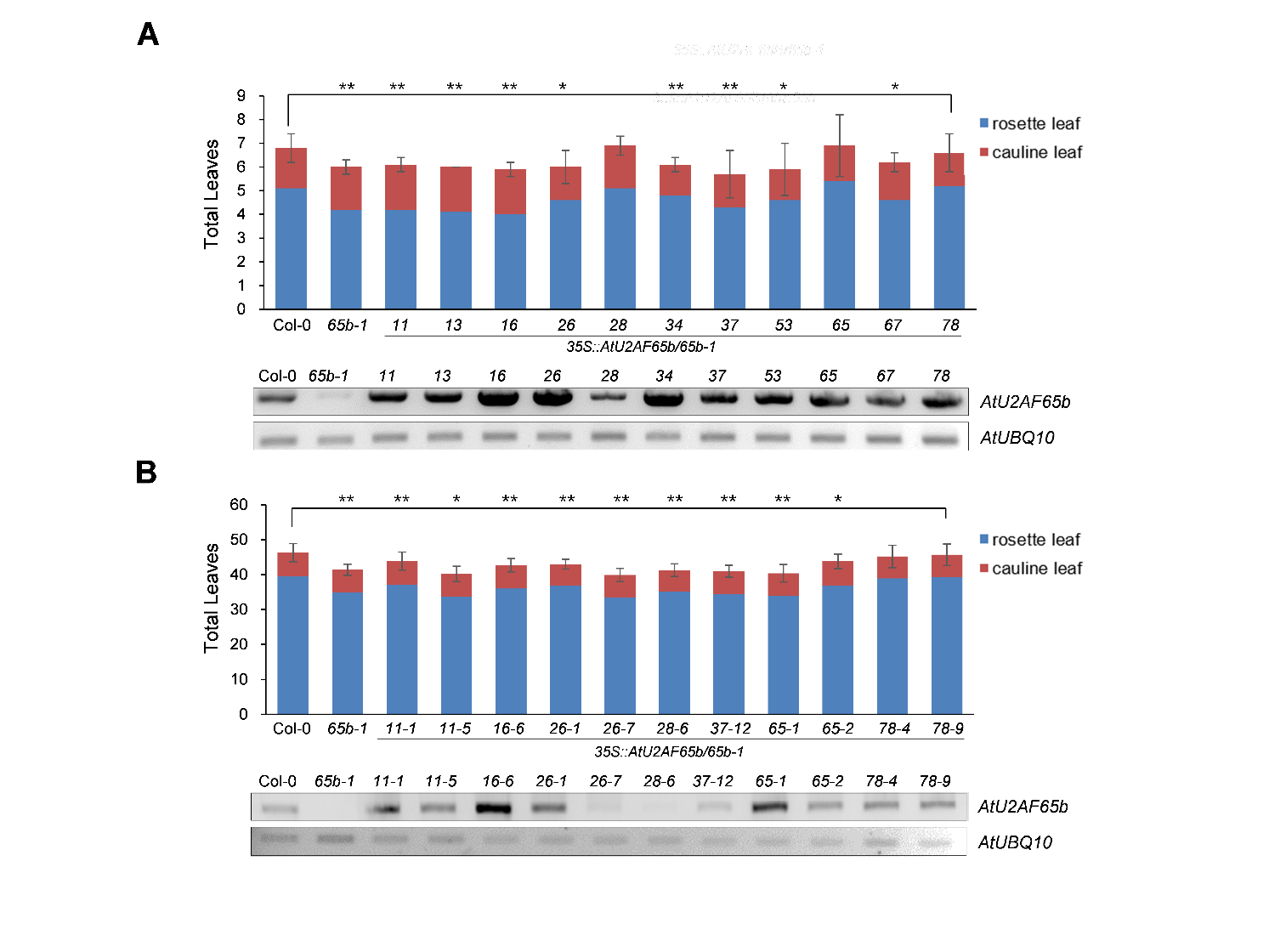


**Figure S2. Flowering phenotype of the rescued transgenic plants of the *atu2af65b* mutants.** The full-length cDNA of *AtU2AF65b* was introduced into the *atu2af65b-1* mutant plants using *35S* promoter (*p35S::AtU2AF65b* in *atu2af65b-1*) to restore the abnormal flowering phenotype of the mutant plants. Plants were grown at 23^o^C under LD (**A**) and SD (**B**) conditions. Flowering time of the transgenic plants in the T_2_ generation was measured. Total leaves (rosette leaves plus cauline leaves) shown in the bar graphs represented the flowering phenotypes of each plants. As a control, the wild-type (Col-0) plants were used. Error bars indicate the standard deviation. The asterisks denote a significant difference in the flowering time of the transgenic plants compared with that of the wild-type (Col-0) plants (Student’s *t*-test, ^*^*P* < 0.05, ^**^*P* < 0.01). Noted that the transgenic lines with *AtU2AF65b* expression except 78-4 and 78-9 did not complement the early flowering phenotype of *atu2af65b-1* mutants.


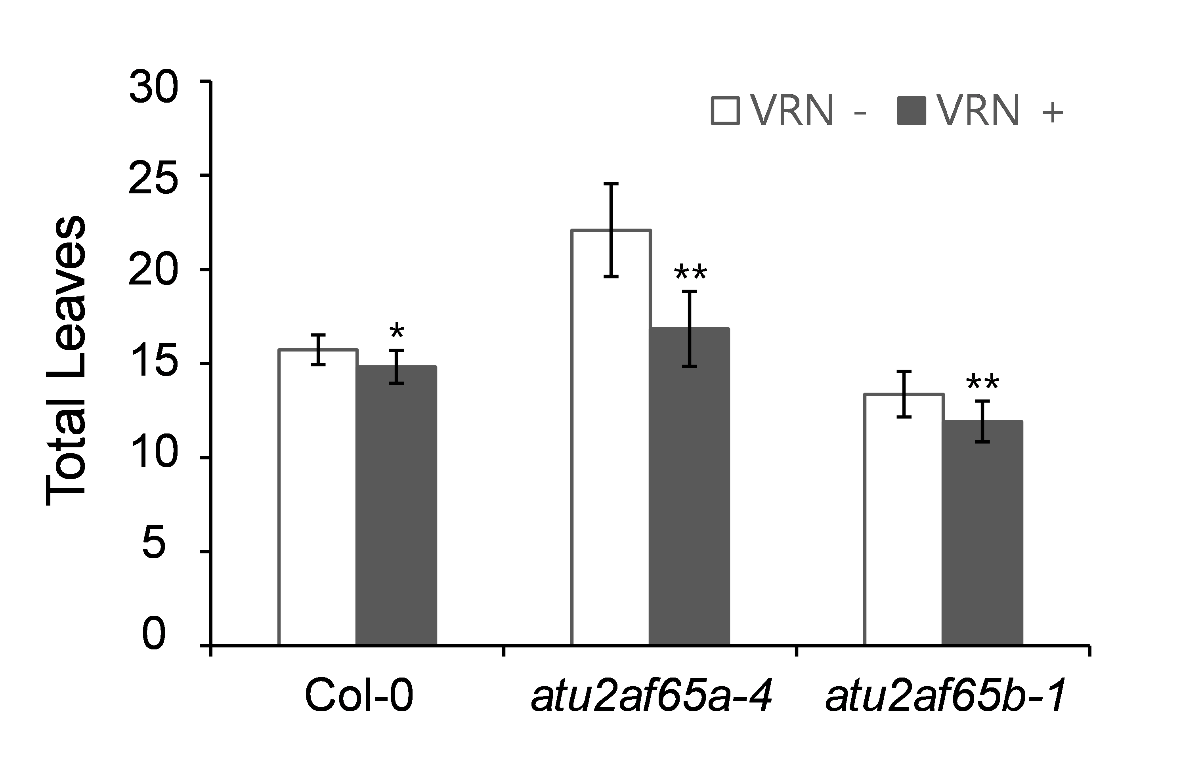


**Figure S3. Effect of vernalization on flowering time of *atu2af65a-4* and *atu2af65b-1* mutants.** Plants were grown at 23^o^C under LD conditions. Error bars indicate the standard deviation. The asterisks denote a significant difference in the flowering time of the transgenic plants compared with that of the wild-type (Col-0) plants (Student’s *t*-test, ^*^*P* < 0.05, ^**^*P* < 0.01).


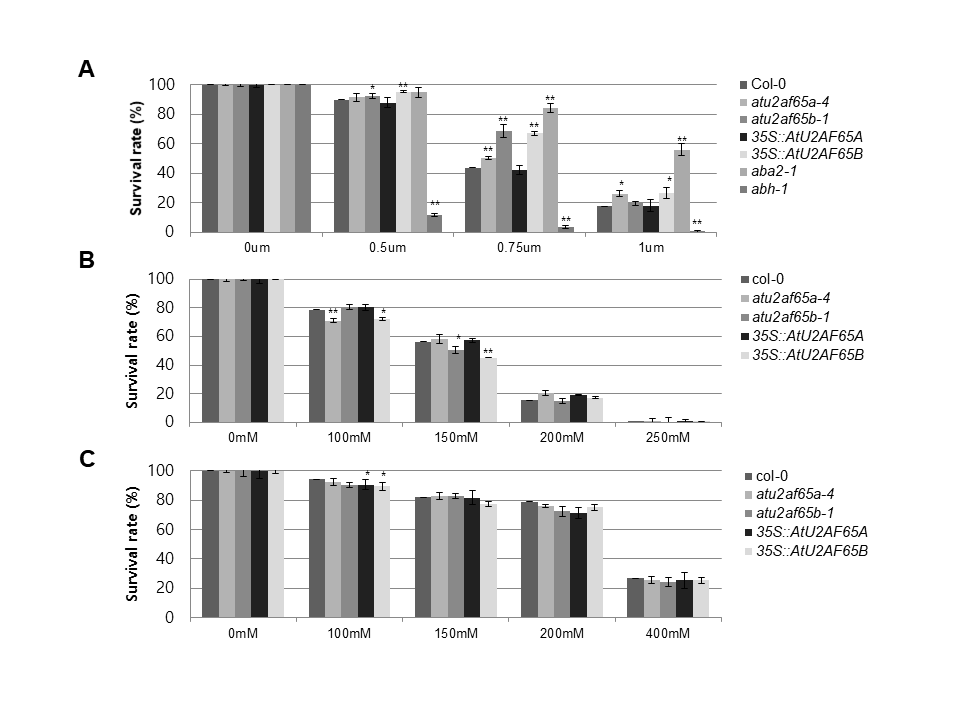


**Figure S4. The effect of ABA, NaCl, or mannitol treatments on seed germination efficiency.** The survival rates of each plants treated with abscisic acid (ABA) (**A**), NaCl (**B**), and mannitol (**C**) were measured at 10 days. The overexpressors (*p35S::AtU2AF65a* and *p35S::AtU2AF65b*), *aba2-1*, *abh-1*, and wild-type (Col-0) plants were used for comparison. Error bars indicate the standard deviation. The asterisks denote a significant difference in the response of abiotic stresses of the plants compared with that of the wild-type (Col-0) plants (Student’s *t*-test, ^*^*P* < 0.05, ^**^*P* < 0.01).


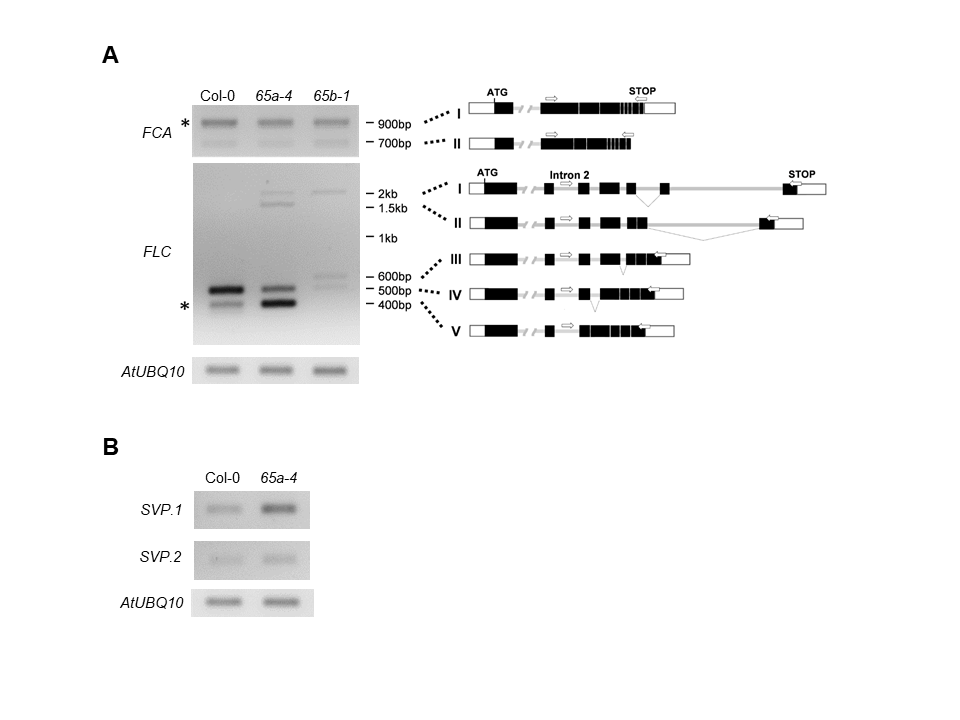


**Figure S5. Effect of *AtU2AF65* mutation on alternative splicing events in *FCA*, *FLC*, and *SVP*.** (**A**) RT-PCR analyses showing detection of spliced or unspliced isoforms of *FCA* and *FLC* transcripts in *atu2af65a-4* and *atu2af65b-1* mutants grown at 23^o^C under LD conditions. In RT-PCR analysis of *FCA*, upper and lower PCR bands show *FCA-γ* and *FCA-δ* transcripts, respectively. In RT-PCR analysis of *FLC*, schematic gene structures of major or alternatively spliced *FLC* transcripts detected are shown. Boxes and lines indicate the exons and introns, respectively. Fully spliced mRNA isoforms are denoted by black lines in *atu2af65a-4* and *atu2af65b-1* mutants. Arrows and asterisks denote the positions of primers and the fully spliced *FCA* and *FLC* transcripts, respectively. *AtUBQ10* expression served as a loading control. (**B**) RT-PCR analysis showing detection of two spliced isoforms (*SVP.1* and *SVP.2*) of *SVP* transcripts in *atu2af65a-4* mutants grown at 23^o^C under LD conditions. *SVP.1* and *SVP.2* transcripts indicate the major and minor spliced *SVP* transcripts, respectively. Note that the expression of major *SVP.1* transcripts was altered in *atu2af65a-4* mutants, whereas minor *SVP.2* transcripts was not.
